# Ocular Drug Delivery: Emerging Approaches and Advances

**DOI:** 10.3390/pharmaceutics17050599

**Published:** 2025-05-01

**Authors:** Shilpkala Gade, Yin So, Deepakkumar Mishra, Shubhamkumar M. Baviskar, Ahmad A. Assiri, Katie Glover, Ravi Sheshala, Lalitkumar K. Vora, Raghu Raj Singh Thakur

**Affiliations:** 1 School of Pharmacy, Queen’s University Belfast, Medical Biology Centre, Belfast BT9 7BL, UK; sgade01@qub.ac.uk (S.G.); yso02@qub.ac.uk (Y.S.); dmishra01@qub.ac.uk (D.M.); sbaviskar01@qub.ac.uk (S.M.B.); aassiri01@qub.ac.uk (A.A.A.); kglover07@qub.ac.uk (K.G.); 2 College of Pharmacy, Najran University, Najran 1988, Saudi Arabia; 3 Department of Pharmaceutics, School of Pharmacy, Anurag University, Hyderabad 500088, Telangana, India; ravi.pharmacy@anurag.edu.in

**Keywords:** ocular disorders, advanced drug delivery systems, sustained drug release, pharmacokinetics, pharmacodynamics

## Abstract

Complex anatomical and physiological barriers make the eye a challenging organ to treat from a drug delivery perspective. Currently available treatment methods (topical eyedrops) for anterior segment diseases pose several limitations in terms of bioavailability and patient compliance. Conventional drug delivery methods to treat posterior segment ocular diseases are primarily intravitreal injection (IVT) of solutions. IVT is highly invasive and leads to retinal toxicity, endophthalmitis, and intraocular inflammation, frequently requiring professional administration and frequent clinical visits. Advanced drug delivery treatment strategies could improve patient compliance and convenience. Long-acting drug delivery platforms (biodegradable or nonbiodegradable) provide sustained/controlled release of drugs for at least four to six months. Smart drug delivery alternatives, for instance, in situ forming implants, are injectable formulations that form semisolid-to-solid implants in response to the various stimuli of pH, light, osmolarity, and temperature. Additionally, nanoparticulate drug delivery systems, contact lenses, electrospun patches, and microneedle-based drug delivery systems provide minimally invasive treatment options for ocular disorders. This comprehensive review focuses on advanced drug delivery options for the management of ocular disorders.

## 1. Introduction

Ocular drug delivery represents a formidable challenge in pharmaceutical sciences due to the eye’s complex anatomy and robust protective mechanisms. Traditional formulations, such as eye drops, have been widely used for anterior segment disorders; however, they are plagued by several inherent limitations. Due to the rapid clearance by tears and blinking, along with barriers posed by the corneal epithelium, these formulations typically achieve less than 5% bioavailability. This low retention time necessitates frequent administration, which can lead to patient non-compliance and suboptimal therapeutic outcomes [1].

For posterior segment disorders, intravitreal injections remain the predominant delivery method. While these injections facilitate higher drug concentrations within the vitreous humor, they are invasive procedures associated with significant risks such as retinal detachment, intraocular inflammation, and endophthalmitis. The repeated administration required to maintain therapeutic levels further increases the risk of complications and poses a considerable burden on both patients and healthcare systems [2,3,4].

**Figure 1:**
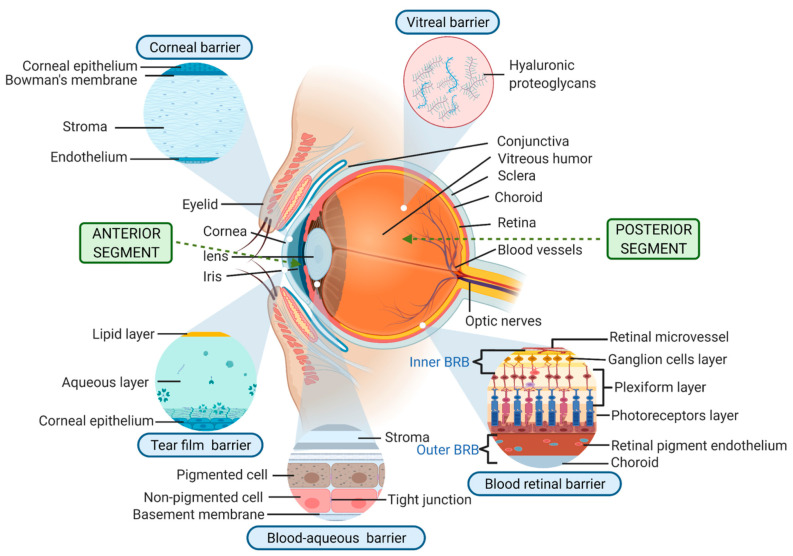
Anatomy of the eye showing the anterior and posterior segments, along with key barriers (tear film, cornea, and blood-retinal barrier) that impede drug delivery [6].

Moreover, the eye’s complex structure with its multiple layers and unique barriers (Figure 1) complicates effective drug penetration, making it challenging to achieve sufficient drug concentrations at the target site. The dynamic environment of the ocular surface, influenced by rapid tear turnover and constant mechanical clearance, further exacerbates these difficulties by reducing the residence time of topical formulations [5].

Addressing these obstacles is imperative for enhancing the treatment of a wide range of ocular diseases. Innovations in formulation and delivery technologies are required to improve bioavailability and extend drug residence time, ultimately reducing the frequency of administration. Such advances have the potential to markedly improve the management of both anterior and posterior segment disorders, resulting in improved therapeutic efficacy and increased patient adherence.

## 2. Advanced Drug Delivery Systems for Ocular Disorders

**Figure 2:**
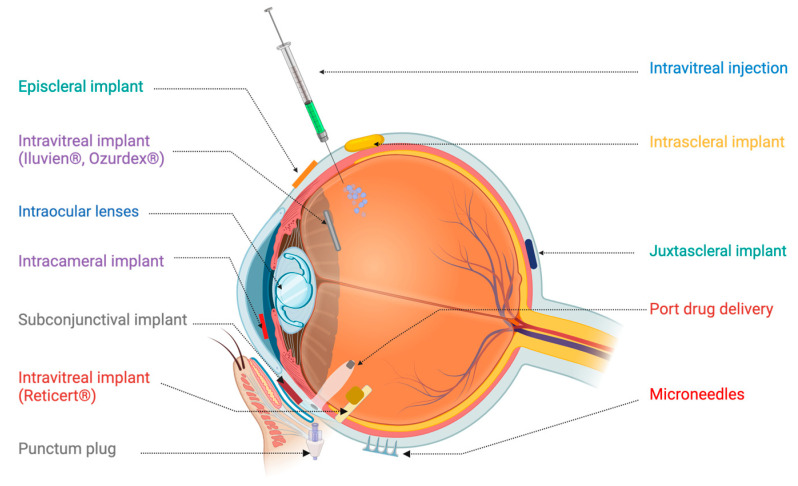
Advanced drug delivery systems for intraocular applications.

Advanced drug delivery systems represent a paradigm shift in the treatment of ocular disorders compared to conventional methods, which are often limited by poor bioavailability and frequent dosing requirements. Advances in material science and biotechnology have catalyzed the development of biodegradable biomaterials that enable the fabrication of a range of sophisticated drug delivery platforms. These include nanoparticles, microparticles, hydrogels, and implantable devices that offer controlled and sustained release of therapeutics over extended periods. Such systems not only improve therapeutic outcomes by maintaining steady drug concentrations but also have the potential to reduce the socioeconomic burden on patients by minimizing the frequency of clinical interventions [7,8,9,10,11,12]. Figure 2 shows the currently available novel drug delivery systems for the management of eye diseases.

Critically, these advanced platforms can be classified into several key categories based on their formulation and delivery mechanisms. Preformed implants—both biodegradable and nonbiodegradable—are designed to achieve long-term drug release through surgical or minimally invasive insertion into ocular tissues. In situ forming implants offer an alternative by transitioning from liquid to solid state upon exposure to specific physiological stimuli (e.g., temperature, pH, or light), eliminating the need for surgical removal post-therapy [13,14]. Electrospun patches, fabricated via electrohydrodynamic atomization, create fibrous scaffolds with high surface areas ideal for tissue interfacing and controlled drug release [15]. Nanoparticulate systems are tailored to enhance drug permeation and retention in ocular tissues, while microneedle-based approaches provide a minimally invasive method for both transdermal and ocular delivery, ensuring precise targeting with reduced tissue trauma. Additionally, drug-eluting contact lenses are emerging as a noninvasive, sustained-release modality particularly suited for anterior segment disorders.

**Figure 3:**
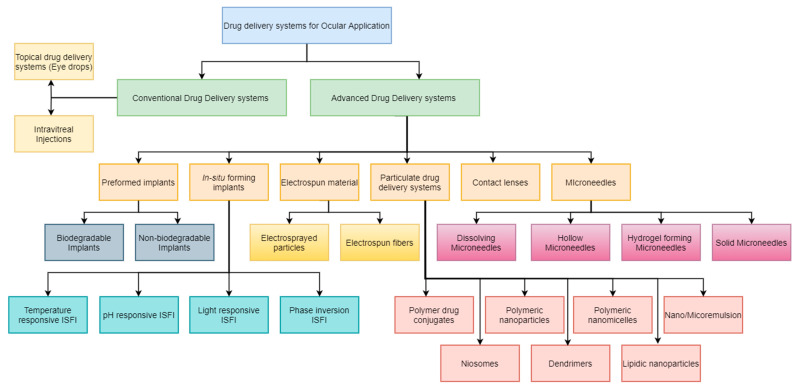
Novel drug delivery system classification for ocular applications.

Looking forward, these technologies collectively promise transformative improvements in ocular therapy. However, challenges remain in terms of optimizing formulation stability, achieving uniform drug release profiles, ensuring biocompatibility, and scaling up manufacturing processes for clinical applications. Continued interdisciplinary research will be essential to address these hurdles and to integrate combinatorial strategies—such as merging nanoparticle carriers with implantable systems—to further refine therapeutic outcomes. As illustrated in Figure 3, the diverse classification of these advanced drug delivery systems underscores their potential to be tailored for specific ocular conditions, thereby paving the way for personalized treatment regimens that can significantly improve patient quality of life.

### 2.1. Preformed Ocular Implants

Preformed implant devices are designed for the sustained release of therapeutic ingredients that are prefabricated and injected with the help of needles or surgery. One of the major advantages of long-acting preformed implants is that they provide convenience to patients in terms of a reduced frequency of administration of conventional eye drops for long-term treatment. Implants for sustained release of protein/peptide and corticosteroid delivery have been widely explored in recent years. A summary of FDA-approved sustained-release implants is shown in Table 1. Neurotech Pharmaceutical developed a semipermeable hollow fiber membrane (NT-503) that encapsulates VEGF receptor Fc-fusion protein (VEGFR-Fc)-expressing cells, which are indicated for the treatment of wet AMD [16]. NT-503 technology provides a platform to release VEGFR-Fc 20 times more efficiently in inhibiting VEGF than ranibizumab and lasts for over two years. The ODTx injectable device is a laser-activated drug delivery system developed by On Demand Therapeutics. The device contains reservoirs that can store both small- and large-molecule drugs and releases the drug by activation of a specific noninvasive ophthalmic laser [17]. Surmodics Inc. developed a sustained-release helical coil implant (I-vation) containing 0.925 µg triamcinolone acetonide coated in PVA, EVA, and titanium. This helical coil design allows it to be secured on the sclera to provide drug delivery for up to 2 years [18]. Phase I trial results proved its effectiveness in the treatment of DME. However, the work was terminated in Phase IIb [19]. DEXYCU^®^ is a dexamethasone 9% intraocular suspension approved in 2018 for the treatment of postoperative inflammation associated with cataracts [20]. Verisome™ technology is a biodegradable (liquid in the aqueous phase) novel sustained-delivery platform investigated by EyePoint Pharmaceuticals for delivering small and large molecules [18]. Cortiject^®^ is an injectable ocular emulsion that employs EyeTech Technology—a system designed to deliver a corticosteroid prodrug, which becomes pharmacologically active upon enzymatic activation within the retina and choroid. This device contains a preservative-free dexamethasone prodrug emulsion, which is further activated by specific enzymes located at the choroid/retina regions, resulting in fewer side effects due to target-specific binding. Cortiject^®^ is injected into the vitreous humor via intravitreal injection, which aims to provide sustained drug delivery for 9 months for the management of DME. Cortiject^®^ is currently in a phase I clinical trial to evaluate its safety and efficacy [21]. Failure of these preformed implants in clinical trials suggests the need for thorough investigation by the PK-PD model. This section mainly focuses on two main types of preformed implants used for ocular delivery: biodegradable and nonbiodegradable implants.

#### Biodegradable Preformed Implants

Biodegradable implants, as the name suggests, are designed to naturally degrade at the administration site; hence, only an initial surgical procedure is needed for the administration of these implants at the target site. However, these are often associated with several drawbacks, such as initial burst release and poor linearity of drug release, restricting their overall effectiveness [22]. Furthermore, release patterns could become unpredictable; therefore, it could be challenging with narrow therapeutic index drugs cyclosporin, amphotericin B, and sunitinib [23]. Biodegradable implants could be composed of synthetic or naturally occurring polymers. Polycaprolactone (PCL), poly(ethylene glycol) diacrylate (PEGDA), poly(lactic-co-glycolic acid) (PLGA), poly(lactic acid) (PLA), poly(glycolic acid) (PGA), and polyvinyl alcohol (PVA) are examples of synthetic polymers. The typical mechanism of the degradation of biodegradable types of implants is hydrolytic cleavage, in which water-soluble and nontoxic byproducts are produced that can be easily eliminated from the site of implantation via blood circulation [24]. PLGA has been extensively used in the fabrication of biodegradable medical devices due to its approved use by the FDA, its safety profile, and, most importantly, its relatively fast degradation process, approximately 4 to 6 months [25].

Ozurdex^®^ by Allergan, a dexamethasone intravitreal implant, was FDA-approved in 2009 for the treatment of adults with macular oedema after branch retinal vein occlusion or central retinal vein occlusion, noninfectious posterior uveitis, and diabetic macular oedema (DMO) [26]. Biodegradable implants are often commonly composed of PLGA and similar polymers, which break down into lactic acid and glycolic acid gradually by hydrolysis, sequentially to carbon dioxide and water; therefore, all byproducts of PLGA can be eliminated from the eye. Ozurdex^®^ shows triphasic drug release, i.e., a high release rate initially for approximately 2 months, then it reaches a plateau for a steady release of dexamethasone for approximately 4 months, followed by a rapid release phase due to hydrolysis [27]. Bhagat et al. conducted a drug release study on intact unfragmented implants and 3-piece implant fragments to study how the fragmentation of implants in the body affects the dissolution/release of dexamethasone from implants. However, data for drug release in rabbits (i.e., concentration of dexamethasone in vitreous humor and aqueous humor) were similar for both the intact and fragmented Ozurdex^®^ implants, suggesting that fragmentation did not affect the dissolution of dexamethasone. The results are in accord with the in vitro reverse engineering study results obtained by Costello et al. 2023 [28,29,30]. Durysta™ (by Allergan, Inc., Dublin, Ireland) is a PLGA-based biodegradable implant injected intracamerally (via a single injection using a preloaded specific applicator with a 28-gauge needle) (approved by the FDA in March 2020) to deliver bimatoprost. Durysta™ provides a pharmacokinetic advantage over topical bimatoprost eye drops by providing sustained release for a period of 3 months to treat ocular hypertension and open-angle glaucoma. The combination of the drug delivery system and route of administration allows for the sustained release of bimatoprost from just 10 µg reservoirs to last for a long time and maintain the therapeutic concentration over several months [31].

Transscleral implants, on the other hand, provide a slightly altered pharmacokinetic profile compared to the in vitro drug release profile. Okabe et al. developed intrascleral implants of betamethasone phosphate (BP). The implants were made up of poly(DL-lactide) with a diameter of 4 mm and a thickness of 0.5 mm. These biodegradable implants illustrated a biphasic drug release pattern in vitro. Furthermore, in vivo pharmacokinetic analysis in rabbits suggests that the highest amount of drug was found in the retina-choroid compared to the vitreous humor. Additionally, below the detection limit, levels of BP were found in aqueous humor, suggesting low permeation of the drug in the anterior segment compared to the posterior segment [32]. The study found altered drug release kinetics in vivo, as no biphasic release was observed in vivo, and only 8% of the drug was released in the first week, with faster drug release at the later stages. This could be attributed to the different sink conditions in vitro and in vivo; furthermore, water absorption is initially low, with increased channel formation in the matrix leading to faster release of betamethasone phosphate; however, the mechanism leading to increased channel formation requires detailed studies to ensure proper understanding. Studies have shown that the sclera is a barrier to drug delivery to the posterior segment of the eye and that drug molecules permeate more through the surgically thinned sclera than through the normal sclera. The diffusion of molecules through the sclera depends on the molecular weight and molecular radius of the molecules [33]. Ambati et al. (2000) demonstrated the possibility of achieving therapeutic concentrations of bioactive protein in the retina choroid of the eye via the transscleral route [34]. Although therapeutic concentrations of drugs are found in the choroid, the retinal pigment epithelium acts as an outermost blood–retinal barrier, forming tight junctions between the RPE and the vitreous humor that are permissible to very limited molecules. A biodegradable intrascleral implant of betamethasone achieved therapeutic drug concentrations in rabbit choroid and retina for up to 8 weeks. The 0.5 × 4 mm (height × diameter) implant was fabricated from PLA containing 25% w/w betamethasone phosphate. The implant was surgically placed in a 100 µm deep intrascleral pocket [32].

### 2.2. Nonbiodegradable Preformed Implants

Nonbiodegradable intraocular implants tend to be more invasive than biodegradable implants, as an additional surgical procedure is needed to retrieve the implant once its drug cargo is completely delivered. Surgical removal of implants could lead to a risk of infection, for example, endophthalmitis. However, such implants tend to demonstrate a long-term zero-order kinetic drug release pattern; hence, the release profiles are more predictable and reliable, and burst release can be controlled [35]. Typical polymers used for composing nondegradable implants include silicone, poly(methyl methacrylate) (PMMA), and ethylene vinyl acetate (EVA).

Ocusert^®^, the first FDA-approved ocular implant, was a nonbiodegradable implant designed to provide sustained release of pilocarpine (to treat glaucoma) and was developed by Alza Corporation in 1974. Ocusert^®^ was composed of EVA and designed to be placed under the lower eyelid. This device aimed to deliver pilocarpine at a rate of 20 to 40 µg per hour for at least a week to replace the application of topical eye drops [36]. Vitrasert^®^ (an antiviral implant indicated for cytomegalovirus (CMV) retinitis linked to AIDs) by Bausch + Lomb is another nonbiodegradable implant that was approved in 1995. The implant was composed of PVA and EVA and was designed to provide sustained release and targeted action of ganciclovir as an alternative to intravenous injections. Each Vitrasert^®^ implant contained 4.5 mg of ganciclovir, which was designed to release the drug for approximately 6 to 8 months.

Fluocinolone acetonide nondegradable ocular implants composed of silicone and PVA were launched by Bausch + Lomb in 2015 (Retisert^®^) and Alimera Sciences in 2011 (Iluvien^®^). Each Retisert^®^ implant contained 590 µg of fluocinolone acetonide and was designed to release the drug over a period of 30 months for the treatment of noninfectious posterior uveitis. The initial release rate was approximately 0.6 µg per day and dropped to a steady 0.3 to 0.4 µg per day over the first month [37]. Each Iluvien^®^ implant contains 190 µg of fluocinolone acetonide and is indicated for DME treatment. This implant is administered into the vitreous humor using a single-use applicator with a 25-gauge needle [38]. Fluocinolone acetonide is released from the implant at a rate of 0.25 µg per day initially and lasts up to 36 months. The sustained release of implants allows the duration of the implant to last long enough to control DME. Therefore, surgical removal of this implant would not be necessary as long as it does not affect the patient’s vision. Hence, this overcomes the drawback of some of the implants discussed earlier. Susvimo™, a refillable port delivery system (PDS) developed by Genentech, was approved by the FDA in October 2021. This PDS is designed to reserve ranibizumab and sustain its release for several months for the treatment of neovascular (wet) age-related macular degeneration (AMD). The intravitreal implant requires one-time surgical insertion into the eye and refilling every six months with an injection containing 100 mg/mL ranibizumab. The phase II LADDER trial has shown that Susvimo™ was as effective as monthly intravitreal injections over 9 months, based on both visual acuity measures (corrected visual acuity) and anatomical measurements (measurements of central foveal thickness) [39]. The approval is based on a succession of Phase III Archway trials, of which Susvimo™ has proven to attain vision gains in wet AMD patients for up to 40 weeks of treatment. Only 1.6% of Susvimo™ patients needed an additional ranibizumab dose before their first refill; hence, it demonstrated its effectiveness for over 98% of wet AMD patients for up to 6 months [40].

### 2.3. In Situ Forming Implants (ISFI)

Some of the conventional implants available for the long-term management of posterior eye diseases such as AMD are performed in vitro and implanted into the eye via surgical methods. Furthermore, removal of nondegradable implants after complete drug release needs to be taken into consideration during the development of such delivery systems. Surgery could be highly invasive and could easily lead to side effects such as increased intraocular pressure, infection, bleeding, or retinal detachment [41]. On the other hand, in situ forming implants (ISFI) would provide advantages over traditional implants in terms of their less invasive nature. The principle of ISFI is to directly inject the drug-loaded gel into the target site and then stimulate it through pH, temperature, or light to initiate the formation of a solid implant. In addition, ISFI made of biodegradable materials such as PLGA can also avoid post drug release surgical operations and complications. Tsujinaka et al. (2020) prepared self-aggregating PLGA microparticles loaded with sunitinib for the management of AMD via intravitreal injections [42]. Currently, it is in a phase II clinical trial (NCT03953079). The PLGA-based in situ sunitinib formulation administered every 6 months is being compared with injections of aflibercept every 2 months in patients with neovascular age-related macular degeneration (abbreviated as the ALTISSIMO study). Various stimuli can be used to form implants in the body. ISFIs are typically classified based on the mechanism triggering their liquid-to-solid transition, such as temperature sensitivity (thermoresponsive), pH change (pH-responsive), light activation (light-responsive), or solvent exchange (phase inversion).

Thermoresponsive ISFIs are drug delivery systems composed of hydrogels that exhibit thermally responsive behavior (sol-gel transition) at the target temperature (usually the physiological temperature of the human body) without additional stimulation to form the implant. Poly(N-isopropylacrylamide) (PNIPAAm), a lower critical solution temperature polymer with a T_g_ of approximately 32 °C, has the potential to form ISFI at body temperature. This property of PNIPAAm-based polymers, i.e., PEGDA-PNIPAAm, has been exploited to develop ISFI systems with sustained release of drugs at the site of administration. A study by Turturro et al. (2018) [43] demonstrated that the PEGDA cross-linked PNIPAAm thermally responsive hydrogel exhibited a decrease in retinal thickness 1 week after injection, as confirmed by OCT imaging. Thermoresponsive hydrogels can also be used to design sustained-release protein formulations. Derwent et al. prepared a poly(N-isopropylacrylamide) crosslinked with a poly(ethylene glycol) diacrylate (PNIPAAm-PEGDA)-based in situ gelling system for the intravitreal drug delivery of proteins [44]. Upper critical solution temperature polymers are another class of thermoresponsive polymers. UCST polymers exhibit a (sol-gel transition or single phase) hydrophobic to hydrophilic transition above the UCST. The mechanism of thermoresponsive behavior normally depends upon destabilization of the electrostatic interaction or hydrogen bonding between the polymer chains. The most widely studied UCST polymer is poly(N-acryloyl glycinamide) (PNAGA), which is the counterpart of the widely explored LCST polymer PNIPAAm [45]. The poly(betaines) also exhibits phase transition behavior due to the temperature-sensitive zwitterionic form [46,47]. The use of UCST polymers is limited in ocular drug delivery due to their wide temperature transition and polymer concentration-dependent behavior. Furthermore, the human body has low temperature tolerance.

pH-responsive ISFIs are generally based on ionizable polymers. The pH-responsive ISFI system usually responds to the pH of ocular fluids, which leads to a change in polymer ionization, initiating the phase transition from the sol to gel state. Pilopine HS^®^ and Timoptic-XE^®^ are two commercially available formulations approved by the FDA that form hydrogel solutions in situ for the treatment of glaucoma [48]. A study conducted by Allam et al. (2019) showed that the pH-responsive natamycin-loaded in situ gel-forming system was more effective than the conventional natamycin formulation due to its antibacterial effect [49]. Studies have shown that the use of a pH-responsive ISFI loaded with betaxolol can effectively control drug release at a release rate of 40.8% over 24 h compared to betaxolol eye drops (89.8% over 24 h). When compared with betaxolol eye drops, the relative bioavailability of pH-responsive ISFI was improved by 280 and 254.7% in normal and glaucomatous rabbits, respectively, and eye retention time was significantly improved due to the conjunction of polymer with mucin [50]. Although pH-responsive ISFI systems have many advantages, the low mechanical stability of such systems potentially affects drug release (Berger et al. 2004) [51]. HPMC has been exploited for its sol-gel transition behavior with respect to pH along with other polymers, including polyacrylic acid, Carbopol^®^ 940, Carbopol^®^ 934 and chitosan [52]. HPMC is a widely used polymer in commercially available topical eye drops and enhances permeation by improving the retention time in the cul-de-sac of the eye.

The light-responsive in situ implant formation system is usually caused by the photoinitiator in the formulation under the stimulation of a certain wavelength of light to cross-link the gel, produce a sol-gel transition, and form a depot implant [53]. The advantage is that due to the light transmittance of the cornea and lens, light can quickly cross-link the gel in a noninvasive manner and demonstrate a low burst release [54]. However, the eye is a delicate organ, and the wavelength of light used for the formation of implants is critical for ocular implants to avoid damaging the eye. A variety of different light sources are used in the photosensitive in situ implant system, such as UV light, visible light, and near-infrared light [55]. However, visible light does not easily control gel cross-linking due to its high exposure intensity and duration, and near-infrared light usually reacts very slowly and requires a long irradiation time, resulting in high burst release. Furthermore, UV-B (280–315 nm) and UV-C (100–280 nm) fail to enter the cornea and are only absorbed on the surface of the cornea. Thus, they are less suitable for the formation of in situ implants [56]. Overall, UV-A (315–400 nm) can penetrate the cornea and lens, reaching the retina, and is hence more suitable for the development of light-responsive intraocular implants [57]. Therefore, UV-A light at 365 nm has been employed for the formation of light-crosslinked implants [58]. UV-A light ensures rapid implant formation, hence reducing the chances of burst release. In addition, the cross-linking speed of the photoresponsive in situ implant system is also affected by photoinitiators. Common photoinitiators used for ocular drug delivery include Irgacure, o-nitrobenzyl, riboflavin, and anthracene [59]. Compared to other photoinitiators, Irgacure 2959 is nontoxic to cells within a certain concentration range and has become a choice of excipient for light-responsive ocular drug delivery [60]. Visudyne^®^ (verteporfin), a light-responsive system, is injected intravenously for the treatment of AMD patients with subfoveal choroidal neovascularization [61]. The subfoeal area (50 J/cm^2^) was illuminated with a light source of 600 mW/cm^2^ with 689 ± 3 nm wavelength light. The subfoveal lesion area is illuminated for 83 s [62,63].

The light-responsive in situ implant formation systems for ocular drug delivery systems involve the use of a photoinitiator in a formulation that, when stimulated by a certain wavelength of light, undergoes a sol-gel transition to form a depot implant. The choice of light wavelength is crucial to avoid any damage to the delicate eye. Different light sources, such as UV light, visible light, and near-infrared light, can be used in these systems. However, UV-A light at 365 nm has been found to be more suitable for the development of light-responsive intraocular implants, as it can penetrate the cornea and lens, reach the retina, and ensure rapid sol-gel transition, reducing the chances of burst release. Photrexa Viscous^®^ is an FDA-approved riboflavin as a photoinitiator to treat keratoconus and corneal ectasia following refractive surgery [64]. Furthermore, a biodegradable light-responsive ISFI system based on PLGA nanomicelles was developed for the intravitreal delivery of peptides [65]. In this study, connexin 43 mimetic peptide (Cx43MP)-loaded PLGA nanoparticles with a size of 149.3–235.4 nm was developed by a two-step nanoprecipitation technique, followed by photocrosslinking of methacrylated alginate. Finally, NPs were incorporated into ISFI for minimally invasive and novel implant systems for intravitreal peptide delivery. Tyagi et al. (2013) [66] developed a gel based on light-sensitive polycaprolactone dimethacrylate (PCM) and hydroxyethyl methacrylate (HEMA) for the delivery of bevacizumab to the suprachoroidal space. In this experiment, PCM was crosslinked with HEMA under 365 nm UV light, and the photoinitiator was 2,2-dimethoxy-2-phenylacetophenone (DMPA). The study demonstrated the controlled release of bevacizumab for up to 4 months in vitro [66].

**Figure 4:**
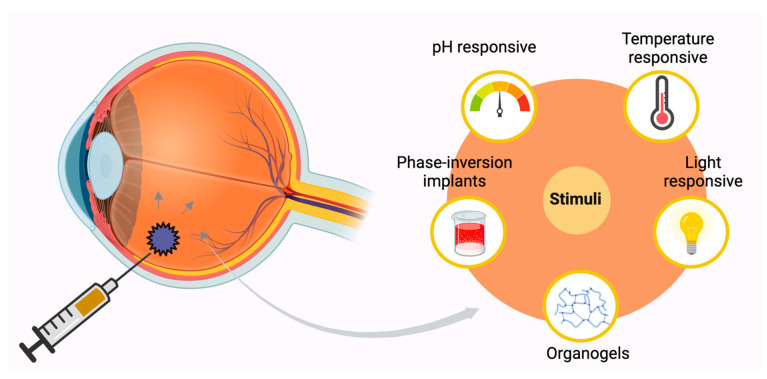
Different types of in situ forming implants.

The concept of phase inversion-based in situ implants was initially introduced by Dunn et al. in 1990, wherein the water-insoluble polymer was dissolved in a mixture of water and water-miscible biocompatible solvent with the therapeutic ingredient [67]. Phase-inversion ISFIs are formed by exposing the hydrophobic polymer contained in the formulation to an aqueous environment to initiate a sol-gel transition to form an implant. This system usually consists of an organic-soluble hydrophobic polymer dissolved in an organic or water-miscible solvent, and the drug is either dissolved or suspended in it. When the system is exposed to the aqueous environment in the vitreous, water enters the system due to diffusion, and the hydrophobic polymer precipitates to form an implant (Figure 4) [68]. The most common FDA-approved polymer for ISFI systems is poly(lactic-co-glycolic) acid (PLGA) [69]. The choice of solvent is also an important factor for phase inversion ISFIs. Usually, owing to their water miscibility, biocompatibility, and certain organic solvents, they are ideal solvents for ISFI formation. Furthermore, the viscosity of the solvent may affect the injectability. The choice of solvents for ISFIs is critical from a biocompatibility point of view. DMSO (dimethyl sulfoxide) [70] and NMP (N-methyl-2-pyrrolidone) [71] are the two commonly used solvents approved for ISFIs. Furthermore, Schoenhammer et al. studied poly(ethylene glycol) 500 dimethylether (PEG500DME) as a solvent for PLGA-based ISFI. The study found that the PEG500DME-based ISFIs were comparatively less hemolytic than PEG600 and NMP [72].

The in situ forming implant system has gained more attention in the field of drug delivery due to its easy injection, low invasiveness, wide compatibility, and patient compliance. However, ISFIs also face some challenges, including burst release during the formation process, which may result in toxicity. Furthermore, solvents used in the formation of ISFIs may result in toxicity depending upon the site of injection; for example, some solvents may cause hemolysis [72]. The rate of drug release is critical for any sustained-release formulation, and the shape of implants may alter the drug release rate. The formation of ISFI is a critical step from the drug release point of view. For example, in ocular drug delivery, implants of different sizes may cause changes in intraocular pressure.

### 2.4. Electrospun Patches

Electrohydrodynamic atomization (EHDA), commonly known as electrospinning, is a versatile technique for fabricating functional polymeric nanofibers with unique morphological features for pharmaceutical applications. In this method, a high voltage is applied to a polymeric solution delivered at a constant rate, generating a charged jet that undergoes stretching and whipping motions before depositing as continuous fibers on a grounded collector. These resulting scaffolds typically exhibit nanoscale diameters, high surface area, and porosity, making them ideal for applications such as wound dressings, tissue engineering, and controlled drug release [73].

**Figure 5:**
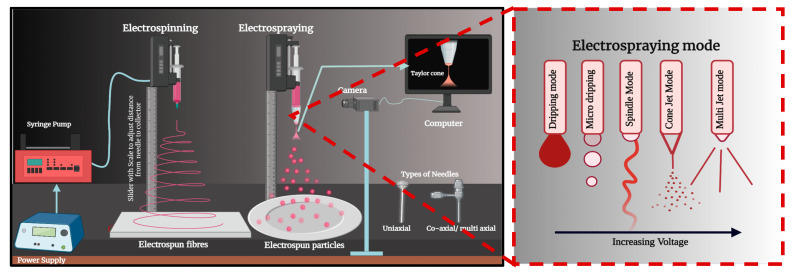
A schematic illustration contrasting electrospinning (**left**) and electrospraying (**right**) for polymer processing. In electrospinning, a high voltage applied to a polymer solution drives a continuous jet that elongates into fibers collected on a grounded substrate. In electrospraying, the same principles direct the liquid into atomized droplets that form particles upon solvent evaporation. Various electrospraying modes (dripping, spindle, cone jet, and multi-jet) emerge by increasing the voltage. The inset highlights the transition from a dripping mode at lower voltage to stable cone-jet and multi-jet modes at higher voltages, each influencing particle or fiber morphology.

Electrospraying, which employs similar equipment and principles, differs primarily in that the polymer solution is broken into smaller droplets rather than forming a continuous jet. The solvent evaporates during flight, leaving behind microscale or nanoscale particles. Adjusting formulation parameters (e.g., drug loading, polymer concentration, and viscosity) and process parameters (e.g., applied voltage, nozzle type, and collector distance) can alter the droplet diameter and yield distinct electrospraying modes such as dripping, spindle, cone jet, or multi-jet, each resulting in different particle morphologies (see Figure 4). While electrospinning typically uses a solution that forms a continuous fiber, electrospraying requires a higher voltage and often a more concentrated polymer solution to achieve the desired droplet breakup (Figure 5) [74,75].

Together, these techniques harness high electric fields to transform polymer solutions into solid structures; their differing mechanisms allow for tailored production of either fibrous scaffolds or fine particles. This adaptability makes them powerful tools for developing advanced drug delivery systems and other biomedical applications.

Andreadi et al. (2022) [76] developed in situ gelling electrospun films of timolol maleate made up of poly(vinyl alcohol) (PVA) and poloxamer 407 for the management of intraocular pressure (IOP). The drug-loaded electrospun fibers were capable of in situ gelling (due to the presence of poloxamer) and prolonged drug release for 24 h [76]. Multilayer electrospinning could be performed to prolong the release of active ingredients. Mirzaeei et al. prepared PVA/chitosan single nanofibers and PVA/chitosan/Eudragit RL 100 multilayer nanofibers for the controlled release of the anti-inflammatory agent ofloxacin [77]. The cylindrical collector was used to collect the nanofibers after electrospinning, and the second layer was coated onto the first layer for slower drug release. The PK analysis of single-layer and multiple-layer electrospun patches shows that the T_max_ is prolonged from 2 hrs to 5 hrs for single-layer and multiple-layer ofloxacin-loaded patches with AUC0-96 values of 3191 ± 117 and 2320 ± 60, respectively, with an enhanced residence time of nearly 18%. Hasbiyani et al. developed collagen with varying amounts of hyaluronic acid and polyethylene oxide-based scaffolds via single-nozzle electrospinning. The uniform nature of electrospun nanofibers and resemblance to corneal surface morphology exhibited high compatibility with the ocular surface [73].

Coaxial electrospinning has been used for the encapsulation of proteins, vaccines, or biomacromolecules to avoid denaturation and aggregation following conventional methods [78]. Angkawinitwong et al. (2017) [79] prepared electrospun fibers of bevacizumab for sustained ocular drug delivery. The electrospun fibers were made up of a poly-e-caprolactone sheath with a core of bevacizumab via the coaxial electrohydrodynamic atomization technique. The prepared 500 nm fibers displayed sustained drug release upon subconjunctival administration. Three-layer nanofibers with an inner and outer layer of gelatin and a middle layer of PCL were successfully prepared with a 25 µm fiber diameter using a multiple nozzle system [80]. Yu (2015) [81] prepared nanofibers made up of ethyl cellulose with varying amounts of ketoprofen in three layers of nanofibers. The prepared nanofibers demonstrated zero-order drug release over a period of 20 h [81].

### 2.5. Particulate Systems in the Management of Ocular Diseases

**Figure 6:**
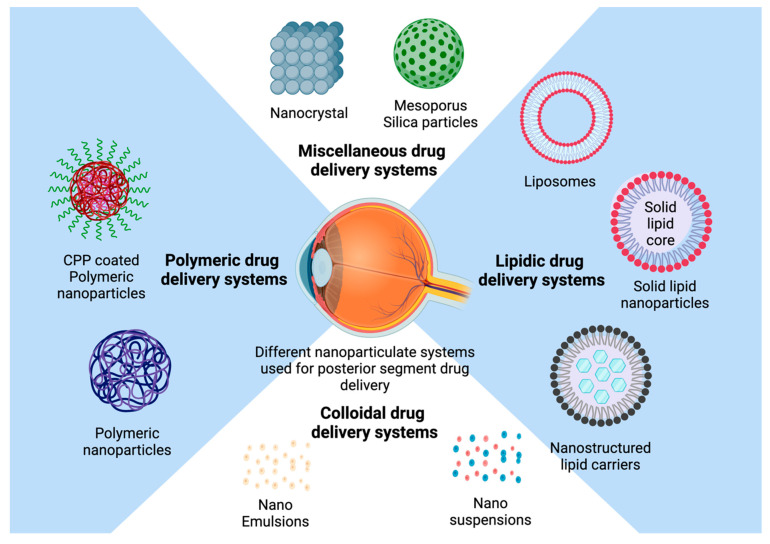
Schematic representation of different types of nanoparticles used in ocular drug delivery systems.

Nanocarriers are differentiated by their fabrication methods, i.e., emulsion, suspension, or compositions, e.g., liposomes and niosomes. The detailed classification of particulate drug delivery systems (DDSs) for ocular application is shown in Figure 6. Various properties of nanocarriers are crucial parameters for fabrication, such as size, polydispersity index (PDI), and zeta potential, which can be tailored to alter the drug distribution and permeation. Owing to their tailorability and advantages over conventional DDSs, various nanocarriers are widely exploited in biomedicine. Table 2 covers the various commercial particulate systems that have been developed for the treatment of ocular disorders and their PK/PD findings. Lin (2016) [82] prepared doxorubicin-loaded hyaluronic acid-modified mucoadhesive liposomes to treat proliferative vitreoretinopathy, a posterior segment eye disease. The liposomes were prepared by the solvent evaporation method, and the surface was modified with hyaluronic acid. An in vivo study on rabbit eyes showed a 1.4-fold higher drug concentration in rabbit aqueous humor compared to free doxorubicin. Liposome drug delivery is a promising tool to enhance the absorption of poorly soluble drugs. However, their short half-life, limited drug loading, and difficulty in sterilization limit the large-scale production of liposomes [82]. Both solid lipid nanoparticles (SLNs) and nanostructured lipid carriers (NLCs), usually containing nanosized lipid cores stabilized by a surfactant layer, have been utilized for loading both lipophilic and hydrophilic drugs inside the lipid core. LNs provide a longer residence time for ocular drugs owing to their mucoadhesive and enhanced cell uptake properties [83]. LNs are exploited in drug delivery due to their controlled release properties, long-term stability for encapsulated drugs, and biocompatibility (as they use physiological lipids), bulk sterilization, and possibility of bulk production.

Particulate systems, including nanoparticles, microparticles, liposomes, dendrimers, and solid lipid carriers, have emerged as promising vehicles for the localized and sustained delivery of chemotherapeutic agents to intraocular tumors such as retinoblastoma and choroidal melanoma. These systems are particularly advantageous in ocular oncology due to their ability to enhance drug solubility, bypass ocular barriers, improve drug retention time, and reduce systemic toxicity.

In the treatment of retinoblastoma, nanoparticle formulations have been designed to enhance the delivery of topoisomerase inhibitors such as topotecan. For instance, topotecan-loaded solid lipid nanoparticles (SLNs) demonstrated enhanced cytotoxicity in retinoblastoma Y79 cell lines compared to free drug, with improved stability and potential for sustained intraocular delivery [84]. Another study used chitosan-coated PLGA nanoparticles for the delivery of carboplatin, which showed enhanced cellular uptake and tumoricidal activity in vitro [85].

Similarly, liposomal formulations have been explored to reduce the dose and frequency of intravitreal chemotherapy. In a rabbit model, liposomal melphalan achieved prolonged vitreal residence and demonstrated potential for minimizing injection frequency while maintaining therapeutic levels [86]. This is particularly relevant for pediatric retinoblastoma patients, where reducing treatment burden and risk of procedural complications is critical.

For choroidal melanoma, particulate carriers have been used to improve the targeting and retention of anticancer agents within the posterior segment. Doxorubicin-loaded PLGA nanoparticles have been shown to localize within uveal melanoma cells in vitro, with enhanced cytotoxicity and reduced drug efflux compared to free drug [87].

Recent work has also explored magnetic nanoparticles as a dual-purpose platform for drug delivery and imaging. These systems can be functionalized with targeting ligands and loaded with chemotherapeutics, allowing for magnetic guidance to the tumor site and potential MRI-based monitoring [88]. Though primarily demonstrated in preclinical settings, this multifunctional approach could enhance both the precision and monitoring of ocular tumor treatments in the future.

Despite their promise, the clinical translation of particulate systems for intraocular tumors remains limited, and ongoing work is focused on optimizing particle size, surface chemistry, and biocompatibility for safe and effective intraocular administration. Nonetheless, the evidence to date supports their therapeutic potential in achieving sustained, targeted treatment with fewer side effects, making them an exciting frontier in ocular oncology.

Qiu (2019) [89] prepared fenofibrate-loaded PLGA nanoparticles for the management of diabetic retinopathy and age-related macular degeneration. Nanoparticle formulations equivalent to 30 µg of fenofibrate were injected into the rat eye via intravitreal injection, and the concentration of fenofibric acid (necessary for activity, formed after the reaction of fenofibrate with esterase found in body tissues) was measured in the vitreous, choroid, retina, and sclera. Subretinal and intraretinal neovascularization was measured using fundus fluorescein angiography. Single intravitreal injections of fenofibrate NPs were sufficient to reduce vascular leakage and choroidal neovascularization by 43% [89]. Triblock copolymers, e.g., PEG-PLGA-PEG, have been found to exhibit improved loading of hydrophobic drugs, such as cyclosorin (CsA). Thommoso et al. (2012) [90] studied the efficacy of cyclosporin-loaded micelles in a corneal graft model. The cyclosporin-loaded micelles showed a 50% reduction in neovascularization compared to saline. Moreover, the treated cornea showed a 73% success rate of corneal graft transplantation. A high CsA value in the cornea of transplanted and healthy rats was observed at 11,710 ± 7530 ng_CsA_/g_tissue_ and 6470 ± 1730 ng_CsA_/g_tissue_, respectively. Significantly lower concentrations were found in the iris-ciliary body, aqueous humor, and vitreous than in the cornea (1570 ± 1080, 770 ± 1510, and 330 ± 220 ng_CsA_/g_tissue_, respectively).

Similar to micelles, niosomes are self-assembled systems; however, they are made up of nonionic surfactants. Unlike liposomes, the main structural component of niosomes is nonionic surfactant, and their size varies from 10 nm to 5000 nm depending on the ratio of surfactant and surfactant/cholesterol. Proniosomal gels loaded with brimonidine niosomes have been found to increase the mean residence time in the cornea by 7.90 times compared to AlphaganP^®^, and the AUC_0-24_ was found to be 5.024 [91]. Dendrimers are multibranched, globular, tree-like nanostructured polymers with a central core and side chains. Drugs are entrapped in either the central core or branch moieties through ionic interactions, hydrogen bonds, or hydrophobic interactions or conjugated through covalent bonds. Dendrimers range in size from nearly 3–10 nm, which is similar to that of small proteins. As opposed to polycationic polymers/particles, dendrimers are noncytotoxic, cleared via urine excretion, and consist of polyamidoamine (PAMAM). PAMAM-based dendrimers were prepared for the intravitreal sustained release of fluocinolone acetonide to treat neuroinflammation in retinal diseases. The study exploited the targeting ability of PAMAM dendrimers to cells with neuroinflammation [92]. Dendrimers can also be used to increase the solubility of drugs with low solubility. For instance, a 2.5 times higher penetration of poorly soluble carteolol from the phosporous-containing dendrimers was achieved compared to carteolol in rabbit eyes. Phosporous-containing dendrimers were synthesized from hexachloro-cyclo(triphosphazene) [93]. Dendrimers have also been utilized for the delivery of microRNAs in vivo. MicroRNAs are 18–25 nucleotide single-stranded small RNAs that function by base pairing with complementary sequences within messenger RNA (mRNA) [94]. The effect of cationic and anionic dendrimers on penetration efficiency in cornea was observed by Souza et al. 2025 [95]. The cationic dendrimer PAMAM 3.5 and anionic dendrimer PAMAM G4 were found to increase the solubility of dexamethasone by 10.3- and 3.9-fold, respectively. The delivery of dexamethasone from the anionic dendrimers showed a 6.6-fold increase in concentration in aqueous humor. This could be due to the sustained delivery of dexamethasone from the anionic dendrimers compared to only 2.5, which was an increase from cationic dendrimers following iontophoresis. This suggests that the PK of the drug can be altered by the selection of a suitable drug delivery system and drug delivery device. In this case, iontophoresis was utilized to enhance drug penetration in the cornea, and the sustained release of dexamethasone led to a higher concentration of drug at the target site [95].

**Table 2:** Commercial particulate systems intended for the treatment of ocular disorders and their PK/PD findings.

Product Name	Drug and Drug Loading	Indication	Type of Formulation	Polymer(s)/Lipids	PK/PD Findings	Route of Administration	Phase of Approval
Restasis^®^	Cyclosporin A	Dry eye syndrome	Nanoemulsion	Castor oil	AUC_0–72 h_ 14,333.2 ng/g.h, corneal clearance of 1.4 g/h [96]	Topical	Approved
Cyclokat^®^	Cyclosporin A	Dry eye syndrome	Cationic nanoemulsion	Castor oil	AUC_0–72 h_ 26,477 ng/g.h Systematic absorption below LOD (0.1 ng/mL) [97]	Topical	Approved
Cequa^®^	Cyclosporin A	keratocon- junctivitis sicca	Nano micellar solution	Octoxynol-40, polyoxyl 40 hydrogenated castor oil	AUC_0–1 h_ 828.25 ± 53.2 ng/g.h [98]	Topical	Approved
Visudyne^®^	Verteporfin	Choroidal neovascularization	Liposomes	EPG and DMPC (3:5 molar ratio)	AUC_0-t_ (µg.h/mL) 1.62, clearance 99.6 [99]	injection	Approved
Lacrisek^®^	Vitamins A and E	Dry eye syndrome	Liposomal spray	Hydrogenated phospholipids	The tear blink intervals were more with Lacrisek^®^ compared to Artelac Rebalance^®^ [100]	Topical	Approved
Artelac Rebalance^®^	Vitamin B12	Dry eye syndrome	Liposomal eye drops	Hyaluronic acid, polyethylene glycol 8000	The aqueous-based Artelac Rebalance^®^ was found to improve TBUT [100]	Topical	Approved
Ikervis^®^	Cyclosporin A	Keratitis in dry eye disease	Cationic nanoemulsion	Medium-chain triglycerides, glycerol, cetalkonium chloride, poloxamer, tyloxapol	AUC_0–72 h_ 26,703.0 ng/g.h, corneal clearance of 0.8 h/h [96]	Topical	Approved
OCS-01	Dexamethasone and cyclodextrin	Postoperative corneal inflammation	Nanoparticles	Cyclodextrin	51% of patients (post cataract surgery) experienced absence of anterior inflammation vs. 19.6% with vehicle control and 72.5% vs. 54.9% with no pain with OCS-01 and vehicle control, respectively [101,102].	Topical	Phase II
SeeQ	CdSe Nanoparticle	Retinitis pigmentosa (RP)	Nanoparticles	Cadmium selenium	BCVA was decreased in patients with RP at least 6 lines in 1 h after IVT [103].	Intravitreal injections	NA
LE-MPP	Loteprednol etabonate	Postoperative inflammation and pain	MPP (Mucus penetrating particles)	Pluronic F127	AUC_0–12 h_ of LE-MPP in cornea and conjunctiva was 1.5-fold higher than lotemax 0.5% eye drops [104]	Topical	Phase III

### 2.6. Drug-Eluting Contact Lenses

Drug-eluting lenses have drawn significant interest in the past decade for sustained ocular drug delivery in a minimally invasive manner [105]. A higher residence time of the drug enhances bioavailability, hence avoiding multiple administrations and leading to higher patient compliance. As a result, the use of contact lenses as drug delivery systems has recently received much attention [106]. They also offer a considerable dose benefit over ocular drops [107]. There are two types of drug-eluting lenses, namely, contact lenses (CLs) and intraocular lenses (IOLs). Contact lenses are further classified into corneal CLs and scleral CLs. CLs can be made up of hydrophilic or hydrophobic polymers and are either hard or soft polymer devices aimed at fitting the cornea to correct refractive errors [108]. IOLs are designed for long-term drug release for chronic conditions. 2-Hydroxyethyl methacrylate (pHEMA) and N-vinylpyrrolidone (NVP) are the most often employed monomers in the production of scleral CLs. These monomers are ideal for usage on the ocular surface because of their high oxygen permeability, high water content, and appropriate wettability [109]. Soft contact lenses have mechanical qualities that allow them to conform to the contour of the eyeball [110].

Drug-loaded contact lenses can be easily manufactured and scaled up using different techniques. The duration of drug release varies depending upon the type of conjugation used for manufacturing drug-loaded contact lenses. For example, ketotifen-loaded presoaked soft contact lenses intended for allergic conjunctivitis were able to sustain drug release for 24 h in the rabbit eye [111]. Lomefloxacin-loaded disposable Acuvue contact lenses were able to maintain the concentration of fluoroquinolone antibiotic above the 90% inhibitory concentration [112]. The contact lenses made up of presoaking the drug were able to maintain the therapeutic concentration in the cornea and aqueous humor for 24 h. Pereira-da-Mota (2021) [113] prepared atorvastatin-loaded contact lenses using molecular imprinting on HEMA hydrogels. The hydrogels were synthesized using different monomers (HEMA, ethylene glycol dimethacrylate (EGDMA), ethylene glycol phenyl ether methacrylate (EGPEM), 2-aminoethyl methacrylate hydrochloride (AEMA) monomers, and atorvastatin. AEMA-imprinted and nonimprinted hydrogels showed significant differences in the drug release pattern of atorvastatin. AEMA hydrogels were found to be promising candidates for atorvastatin-loaded contact lenses that control drug release for approximately 24 h [113].

The incorporation of functional molecules in contact lenses has proven to be a prospective strategy for increasing drug loading in CLs. The potential functional molecules include cyclodextrins, vitamin E, surfactants, and functional monomers. The hydrophobic cavity in cyclodextrins (CDs) provides a suitable site for the incorporation of hydrophobic molecules and has been successfully incorporated as a copolymer in lens backbone materials. Xiao Li et al. prepared poly(2-hydroxyethyl methacrylate-co-methyl methacrylate) (p(HEMA-co-MMA)) hydrogel-based soft CLs crosslinked with beta CDs for sustained delivery of an anti-inflammatory drug (dexamethasone) after cataract surgery [114]. The dexamethasone-loaded contact lenses were able to sustain drug release in vitro for 10 days. The functional monomer (beta CD) on the polymer backbone can control the drug release, and burst release was reduced with respect to an increase in the concentration of CD. In another study, Bouledjouidja et al. loaded ciprofloxacin and dexamethasone 21-phosphate disodium via supercritical CO_2_ impregnation on a foldable IOL made up of poly-2-hydroxyethyl methacrylate (P-HEMA). The impregnation of dexamethasone was higher than that of ciprofloxacin in HEMA contact lenses, which could be attributed to the higher affinity of dexamethasone to HEMA, and the presence of a cosolvent (ethanol) was also responsible for the higher impregnation of dexamethasone compared with ciprofloxacin, as ethanol promotes the solubility of polar drugs. Supercritical fluid is a better solvent for drugs due to its lower surface tension and is an efficient plasticizer for polymers. Moreover, CO_2_ is removed from the CLs or IOLs without an additional purification step and is hence also known as the “green” alternative to organic solvents [115]. However, drug-loaded contact lenses suffer from several drawbacks, namely, the risk of dry eye disease, the threat of infection when incorrectly applied, and concerns about eventual changes in critical properties of the cornea, such as lower water content, poor tensile strength, reduced ion and oxygen permeability, and impaired transparency. Additionally, legal development difficulties and regulatory requirements hinder the dynamic speed of development of CLs as medical devices [105].

### 2.7. Microneedles

Microneedles (MNs) are defined as advanced micron-sized arrays of needles with lengths ranging from 50 µm to 2 mm and external diameters not more than 300 µm [116]. Hashmi et al. (1995) reported the fabrication of the first solid microneedle array using silicon (via wet etching) [117]; however, many materials were later reported, including stainless steel, dextrin, ceramic, maltose, galactose, and various polymers. They are either attached to a syringe or a patch, and owing to their micron size, they can enter the tissue but avoid contact with nerve endings, eventually reducing the pain response. They offer several advantages over conventional needles. They are painless, less invasive, allow faster healing of the applied area, and are more patient compliant and self-administrable. Although conventional hypodermic, intravenous, and intradermal needles provide maximum bioavailability, they are plagued with pain, systemic infection, the need for professionals, and the generation of biohazards. MNs are inspired by hypodermic needles, wherein painless delivery is achieved with minimum generation of waste and needless professional training. In 1998, Mark Prausnitz et al. developed MNs using a reactive ion etching microfabrication technique for transdermal drug delivery, which increased delivery efficacy by 400% [118]. Ever since the introduction of MNs, their application has been explored in a wide range of drug delivery areas, including ocular tissue, including the anterior segment [119] and posterior segment [120,121], vascular tissue [122], the gastrointestinal tract [123], the oral cavity [124], the genitourinary system, and cardiac muscle [125], for therapeutic cell delivery. Microneedles have made it accessible to deliver therapeutics to the less accessible areas of the eye to facilitate the entry of monoclonal antibodies (mAbs) for the management of neovascular diseases.

**Figure 7:**
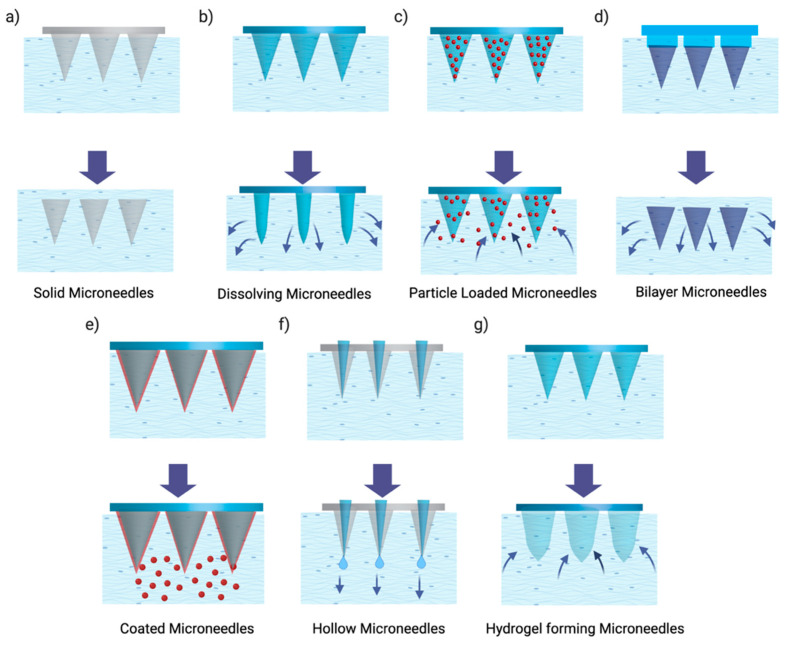
Mechanism of drug release from different types of microneedles. (**a**) Solid microneedles pierce the tissue to create microchannels for drug application. (**b**) Dissolving microneedles release the drug as the needle matrix dissolves. (**c**) Particle-loaded microneedles contain drug-loaded particles within the needle structure. (**d**) Bilayer microneedles consist of two layers for controlled or sequential drug release. (**e**) Coated microneedles have drug coated on their surface which dissolves after insertion. (**f**) Hollow microneedles deliver drug through a central channel. (**g**) Hydrogel-forming microneedles swell in the tissue to enable sustained drug diffusion.

Figure 7 illustrates various kinds of MNs, namely, solid, dissolving, particle-loaded, bilayer, coated, hollow, and hydrogel-forming MNs. MNs have been studied extensively for transdermal drug delivery in the past two decades. Various kinds of MNs have been developed, including simple, for instance, biodegradable, hollow, solid MNs, and more complex, such as detachable and 4D-printed microneedle arrays with backwards-facing barbs [126]. Their low production cost makes them a promising tool for drug delivery in the future. Solid MNs are usually prepared from metals or silicon by dry or wet etching methods. Solid microneedles usually require two-step applications wherein, at first, microchannels are created using MNs, and later, the formulation is applied as a form of patch on the affected area. The coated solid microneedles allow for one-step delivery and use the dip coating technique to coat the microneedle tips with protein-, DNA-, plasmid-, or nanoparticle-containing formulations. Dissolving MNs are made up of biodegradable polymers that are capable of dissolution or degradation in the body. Dissolving microneedles are a promising drug delivery method because they offer higher loading capacity than coated microneedles. Because of the aforementioned MN properties, the site of application is limited to certain areas of the body, such as the arms or abdomen. Furthermore, the dissolution time of dissolving MNs is critical for complete drug delivery.

Dissolving MNs have emerged as a promising platform for ocular drug delivery by leveraging biodegradable polymers that dissolve rapidly upon insertion into ocular tissues. This mechanism allows for high drug loading and efficient release while minimizing invasiveness and patient discomfort. Notably, our team has demonstrated the potential of these systems: rapidly dissolving bilayer microneedles enable efficient protein delivery to the posterior segment [127], and nanosuspension-loaded dissolving bilayer MNs have been effectively applied for hydrophobic drug delivery [128]. In addition, long-acting nanoparticle-loaded bilayer microneedles have been shown to sustain protein release in the posterior segment [129]. Lastly, the application of dissolving MN loaded with deferasirox nanosuspension has also been reported for ocular drug delivery, underscoring the broad utility of this approach in addressing ocular therapeutic challenges [130]. Further details, refer to comprehensive reviews by our group on advanced ocular drug delivery using dissolving MN [131].

Song et al. developed a spring-loaded MN applicator for corneal application. A 140 µm long solid microneedle was attached to a microneedle pen and was found to effectively deliver the rhodamine dye to a 1000 µm^2^ area. Additionally, microneedles were able to deliver an effective volume of sunitinib malate to the suture-induced neovascularization model in vivo compared to the 30G needle tip dipped model [132]. Solid MNs are coated with a polymeric solution or suspension of drug to deliver the drug to the targeted tissue [133]. Drug is released via subsequent dissolution or diffusion through the coated layer. The total amount of drug that can be loaded depends on the thickness of the coated layer and the microneedle size; hence, a low amount of drug can be loaded in coated MNs. Individually coated MNs are used to load different drugs for codelivery. Li et al. 2018 [7] showed that individually coated MNs were capable of delivering multiple compounds to porcine skin. Jiang et al. studied the delivery of drugs to the cornea and sclera via coated microneedles. A solid microneedle with a length of 500–750 µm was coated with protein and DNA. Delivery of fluorescent molecules to in vivo rabbit corneas found that the fluorescein concentration in the aqueous humor was 60 times higher than that in the topical application (without MNs). Another study by Kim et al. targeted the delivery of a large anti-VEGF molecule, bevacizumab, for the treatment of corneal neovascularization. Solid microneedles 400 µm in length with a 55° tip angle were prepared from stainless steel sheets and an infrared laser. A coating solution was prepared by mixing 5% carboxymethylcellulose and dispersing bevacizumab as the active ingredient. Microneedles were coated by dipping them 40–50 times in coating solution. The study found that the microneedles were capable of targeted delivery to the corneal stroma and were able to deliver therapeutic concentrations in a suture-induced corneal neovascularization model [120].

Dissolving MNs are made using biodegradable GRAS (generally recognized as safe) polymers after encapsulating the drug into the polymer. These microneedles dissolve and release the drug after insertion into the ocular tissues and hence do not require removal after insertion. The biocompatibility and biodegradability of these MNs make them one of the best choices for long-term therapy and better patient compliance [119,129]. However, limited drug loading in these types of MNs makes them challenging for ocular applications.

Other types of MNs, such as hydrogel-forming and particle-loaded MNs, are popular for long-acting ocular drug delivery. Lee et al. developed a detachable microneedle with a porous water-soluble layer for ocular delivery. The microneedle consisted of a drug tip and a sacrificial layer made up of poly(vinyl alcohol) (PVA) and poly(vinyl pyrrolidone) (PVP). The effect of the freezing method and polymer concentration on pore formation was studied. Microneedles were frozen by dipping them in liquid nitrogen and by freezing them in a refrigerator. Larger pores were observed in the refrigerator method, which could be attributed to the slow freezing technique. Rapidly detaching microneedles were capable of separation from the sacrificial layer within 2 secs of application [134]. Amer et al. (2020) [135] developed a PVA-based hydrogel forming microneedles in a curved form for ocular application. Microneedles were made with varying heights and lengths in the ranges of 1.8–2.3 mm and 3.35–7.61 mm, respectively. These microneedles were shown to control the release of immunoglobin G1 for 4 weeks compared to a single intravitreal injection of drug solution using a 31G needle. Ovalbumin-PLGA nanoparticle-loaded dissolvable MNs were fabricated using two different molecular weight PVAs (12–23 kDa and 31–50 kDa) by the water-in-oil-in-water emulsion solvent evaporation technique. Multiphoton images of the sclera confirmed the localization of ovalbumin in the sclera in transscleral permeation studies for up to 24 h [129]. The in vitro drug release studies suggested that the particle-loaded bilayer MNs are capable of sustained release of proteins for two months [129].

Hollow MNs have also gained substantial popularity in ocular drug delivery in recent years due to their ability to enable precise and sustained release of therapeutic agents via a minimally invasive approach. These devices enhance drug targeting in both anterior and posterior segments while reducing tissue trauma compared to conventional intravitreal injections. Notably, the proprietary SCS microinjector device has demonstrated successful delivery of a triamcinolone acetonide (Xipere^®^) suspension for the treatment of panuveitis. In addition, clinical trials under the PEACHTREE study are ongoing for diabetic macular edema. For a more detailed discussion on hollow MN technologies in ocular applications, please refer to our comprehensive review by our team [136]. 

## 3. Conclusions and Future Perspectives

Advanced ocular drug delivery systems have demonstrated significant promise in improving therapeutic efficacy by providing sustained release, enhanced bioavailability, and reduced dosing frequency. These innovations, including preformed implants, in situ forming systems, nanoparticulate carriers, and microneedle platforms, have the potential to revolutionize the management of both anterior and posterior ocular disorders, ultimately enhancing patient compliance and quality of life.

The integration of these technologies has already resulted in notable effects, such as improved tissue targeting and minimized invasiveness compared to conventional methods. For example, innovations in microneedle-based systems have enabled precise and minimally invasive delivery to ocular tissues, while nanoparticulate strategies have enhanced drug retention and permeation.

Despite these advances, several challenges remain. Key hurdles include optimizing formulation stability, ensuring consistent drug release kinetics, addressing biocompatibility issues, and developing scalable manufacturing processes. Future research should focus on overcoming these barriers through multidisciplinary approaches, including the refinement of delivery device design and the exploration of combination strategies, to fully translate these promising technologies into clinical practice.

## Figures and Tables

**Table 1:** Overview of commercially available ocular implants.

Device	Medication	Therapeutic Use	Dose	Delivery Route	Release Duration	Composition	Category	Approval Year	Company
Retisert^®^	Fluocinolone acetonide	Non-infectious posterior uveitis	0.59 mg	Intravitreal	30 months	Silicone and polyvinyl alcohol	Non-degradable	2005	Bausch + Lomb (Bridgewater, NJ, USA)
Ozurdex^®^	Dexamethasone	Diabetic macular edema, RVO, uveitis	0.7 mg	Intravitreal	Approximately 6 months	PLGA	Biodegradable	2009	AbbVie (Chicago, IL, USA; Berkshire, UK)
Iluvien^®^	Fluocinolone acetonide	Chronic diabetic macular edema	0.19 mg	Intravitreal	36 months	Polyimide tube with PVA and silicone	Non-degradable	2011	Alimera Sciences, Inc. (Alpharetta, GA, USA; Dublin, Ireland)
Yutiq^®^	Fluocinolone acetonide	Non-infectious posterior uveitis	0.18 mg	Intravitreal	36 months	Polyimide	Non-degradable	2018	EyePoint Pharmaceuticals, Inc. (Watertown, MA, USA)
Durysta^®^	Bimatoprost	Reduction of intraocular pressure	10 mcg	Intracameral	Several months	PLGA combined with PDLA, PDLLA, and PEG3350	Biodegradable	2020	AbbVie (Chicago, IL, USA)
Susvimo^®^	Ranibizumab	AMD	2 mg	Intravitreal	Approximately 6 months	Port delivery system	Non-degradable	2021	Roche (Atlanta, GA, USA)
iDose TR^®^	Travoprost	Reduction of intraocular pressure	75 mcg	Intracameral	3 months	Titanium reservoir with a semipermeable membrane	Non-degradable	2023	Glaukos Corporation (San Clemente, CA, USA)
